# Atypical Distribution of Late Gadolinium Enhancement of the Left Ventricle on Cardiac Magnetic Resonance in Classical Anderson-Fabry Disease

**DOI:** 10.5334/jbr-btr.906

**Published:** 2016-01-29

**Authors:** Shusuke Kasuya, Masayo Suzuki, Tsutomu Inaoka, Masayuki Odashima, Tomoya Nakatsuka, Rumiko Ishikawa, Wataru Tokuyama, Hitoshi Terada

**Affiliations:** 1 Department of Radiology, Toho University Sakura Medical Center, Sakura, Japan; 2 Department of Internal Medicine, Toho University Sakura Medical Center, Sakura, Japan; 3 Division of Surgical Pathology, Toho Univeristy Sakura Medical Center, Sakura, Japan

**Keywords:** Anderson-Fabry disease (AFD), cardiac magnetic resonance (CMR), late gadolinium enhancement (LGE), hypertrophic cardiomyopathy

## Abstract

Anderson-Fabry disease (AFD) is an X-linked lysosomal storage disorder caused by a deficiency of alpha-galactosidase A. Approximately 50% of patients with AFD may have cardiac involvement. Gadolinium-enhanced cardiac magnetic resonance (CMR) is useful for the diagnosis of cardiac involvement of AFD by recognizing typical late gadolinium enhancement (LGE) patterns. We report a 48-year-old man with cardiac involvement in classical AFD, showing atypical distribution of the LGE at the mid-lateral wall of left ventricle, predominantly apical segments without basal involvement on gadolinium-enhanced CMR.

## Introduction

Anderson-Fabry disease (AFD) is an X-linked lysosomal storage disorder caused by a deficiency of alpha-galactosidase A. The estimated birth prevalence of AFD ranges from 1:40,000 to 1:117,000 worldwide. Approximately 50% of patients with AFD may have cardiac involvement, and malignant arrhythmias are the predominant cause of the substantially increased morbidity and reduced life expectancy. Currently, intravenous enzyme replacement therapy is believed to be effective in slowing and halting disease progression, although the success of therapy depends on the stage of the disease [1]. Therefore, the accurate and early diagnosis of AFD is critical for the success of therapy.

Moon JC et al. [2] first reported that cardiac magnetic resonance (CMR) may be useful for the diagnosis of cardiac involvement of AFD by recognizing late gadolinium enhancement (LGE) in the left ventricular (LV) wall. They histologically proved that LGE might be caused by focal myocardial collagen scarring (fibrosis) in AFD [3]. Therefore, CMR has been used to screen for cardiac AFD [4]. De Cobelli F et al. [5] reported the usefulness of gadolinium-enhanced CMR to differentiate cardiac AFD from symmetric hypertrophic cardiomyopathy, assuming the LGE at the inferolateral basal or mid-basal segments of the lateral wall of LV with a mid-myocardial distribution, sparring the subendocardium was specific for AFD. We experienced a case of atypical distribution of LGE at the mid-lateral wall, predominantly apical segments without basal involvement in a patient with classical AFD. Herein we present this case.

## Case report

**Figure 1:**
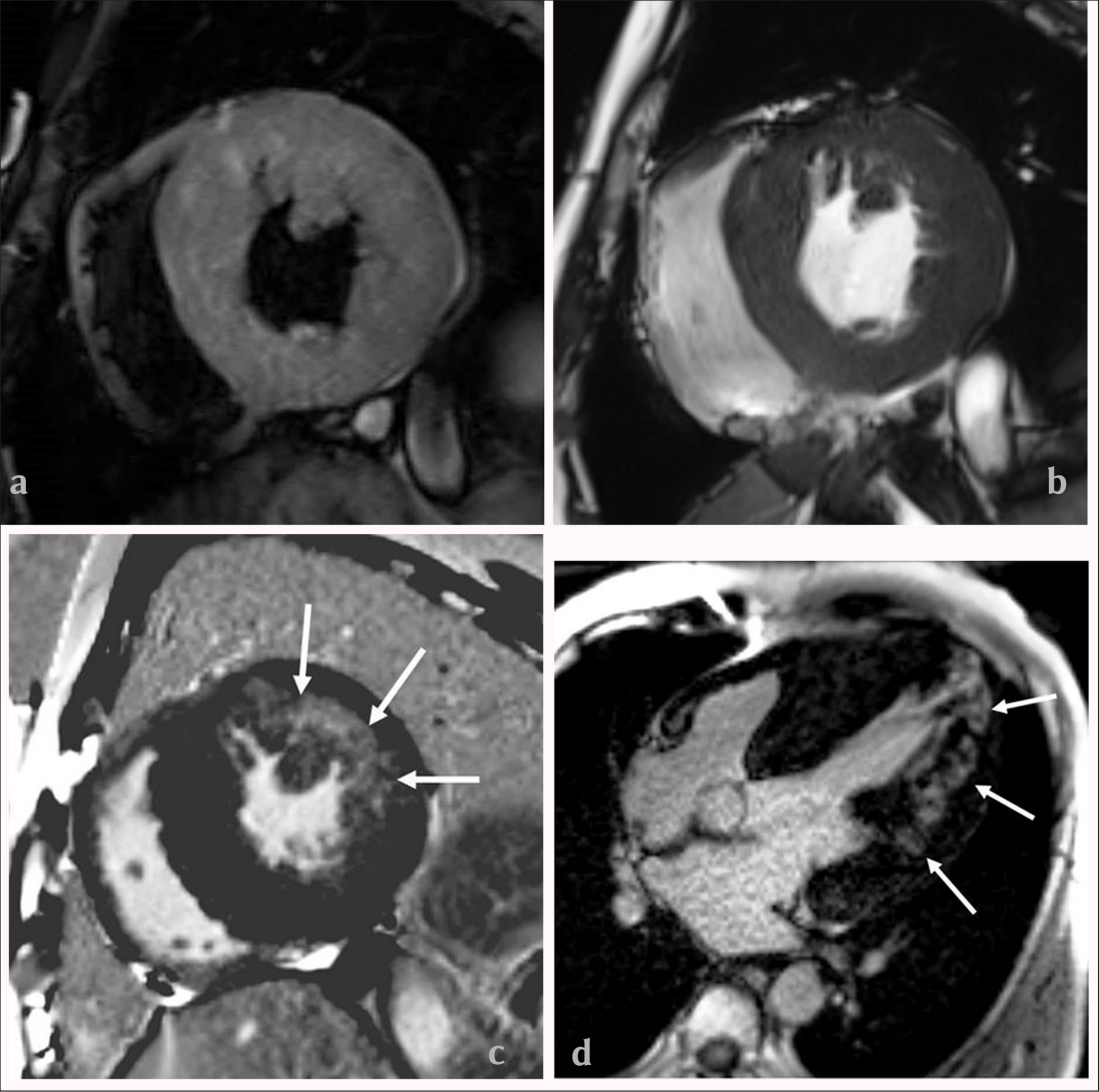
(a) T2WI-STIR with a black-blood technique and (b) short-axis cine image show symmetric hypertrophy of the LV and moderate thickening of the right ventricle. (c) The short axis image shows late gadolinium enhancement (LGE) of the mid-segment of the lateral wall of LV (arrows). (d) The long axis image shows LGE of the mid- and apical segments of the lateral wall of LV (arrows).

A 48-year-old man who had been aware of exertional breathlessness for two years was referred to our hospital because an echocardiogram (ECG) of his medical checkup was abnormal. His medical history included juvenile onset asthma and deafness. Regarding his familial medical history, his paternal grandfather had asthma, his mother had deafness and died from a stroke, and his older sister had asthma. He presented with hypertension and apical systolic murmur. Laboratory data showed moderate renal failure (eGFR: 41 ml/min/1.73 m^2^), high serum level of brain natriuretic peptide (1023 pg/ml), and positive urinary protein. Chest radiography showed only mild cardiomegaly. ECG showed R-wave elevation and T-wave strain abnormalities, and transthoracic echocardiography showed symmetric hypertrophy of the LV (left atrial dimension: 51 mm, interventricular septum thickness: 18 mm, posterior LV wall thickness: 22 mm, ejection fraction: 68%). CMR was performed with a 3 T MR scanner (Magnetom Skyra, Siemens Medical Solution, Enlargen, Germany). T2WI-STIR with a black-blood technique (Fig. 1a) showed symmetric hypertrophy of the LV and moderate thickening of the right ventricle. Short- and long-axis cine images showed hypokinetic wall motion of the LV (Fig. 1b). CMR showed LGE at the mid-myocardium of the mid-lateral through apical segments of LV (Figs. 1c and 1d). Because of the atypical distribution of the LGE of the LV, coronary angiography was performed and showed normal results. An endomyocardial biopsy was also performed and the specimen demonstrated cytoplasmic vacuolation. Alpha-galactosidase activity in the peripheral blood was deficient, and gene mutation analysis showed a homozygote L19p gene mutation. Therefore, a diagnosis of classical AFD was made. He was treated with enzyme replacement therapy.

## Discussion

AFD is divided into “classical” and “non-classical” forms, with classical AFD patients having no residual alpha-galactosidase A activity. Their first clinical symptoms usually arise in childhood between the ages of 3 and 10 years. In general, significant renal, cardiac and cerebrovascular diseases develop after the age of 20 years [1]. The deficiency of alpha-galactosidase A leads to the storage of neutral glycosphingolipids within lysosomes in a variety of cell types. In the heart, coronary microvascular function is actually abnormal, and myocardial perfusion is significantly reduced [1]. Progressive expansion of the cardiac interstitium and myocardial fibrosis develop with age. In fact, the accumulation of glycosphingolipids occurs in all cellular components including cardiomyocytes, conduction system cells, valvular fibroblasts, endothelial cells, and vascular smooth-muscle cells. Histologically, cardiac AFD is characterized by cardiomyocyte hypertrophy and vacuolation. However, in the heart, this accumulation of complex lipids accounts for only 1–2% of the total cardiac mass, pointing towards other mechanisms of disease such as the activation of signaling pathways that result in hypertrophy, apoptosis, necrosis, and fibrosis [1,2].

CMR is a useful tool with which to screen for patients with cardiac AFD as well as those with other non-ischemic cardiac diseases, since the clinical symptoms and examination results, except for those of endomyocardial biopsy, are similar. LGE-CMR is valuable for differentiating AFD from other diseases causing LV hypertrophy. However, the use of gadolinium should be of particular concern in AFD patients because they may have renal failure. AFD patients commonly show LGE at the inferolateral basal or mid-basal segments of the LV lateral wall with a mid-cardial distribution sparing the subendocardium. LGE has been histologically proven to represent areas of myocardial collagen scarring (fibrosis) [2,3,4,5]. Interestingly, the end-stage of cardiac AFD is characterized by intramural replacement fibrosis limited to the basal inferolateral wall of LV [2]. However, the mechanism of distribution remains unknown. It has been speculated that cardiac AFD might predispose the heart to sub-clinical myocarditis leading to tissue injury, or it could impair myocardial resistance to physical stress that might be most prominent in this region where significant shear forces combine with a watershed vascular territory [3,5]. In our case, the reason that LGE was distributed at the mid-lateral and apical segments of LV was unclear. With the progression of AFD, the wider extent of LGE in the LV lateral wall is well known, but the fact that there was no LGE at the cardiac basal segment could not be explained. On the basis of the finding of the mid-cardial distribution at the lateral wall of LV, cardiac AFD was strongly suspected, but cardiac angiography and endocardial biopsy were performed because of the atypical extent from the mid-lateral through apical segments of LGE.

As in other MRI techniques, non-contrast-enhanced T1 and T2 measurement have been reported to be able to differentiate AFD from other diseases with LV hypertrophy such as hypertrophic cardiomyopathy, amyloidosis, and hypertensive disease by means of the measurement of intramyocardial glycosphingolipid accumulation [3,6,7]. In cardiac AFD, T1 mapping may show shorter T1 because of the presence of fat [7]. Since there was no software for analyses of T1 and T2 measurement in our hospital, these measurements were not available.

In conclusion, we reported a case of cardiac involvement in classic AFD with atypical distribution of LGE of the LV on CMR. The reason for the atypical distribution of LGE was unclear. However, while the possibility of cardiac AFD can be suggested by a patient’s clinical symptoms and medical history, it can also be indicated by the mid-cardial distribution of the lateral wall of LV on CMR, even though this distribution may be atypical.

## Competing Interests

The authors declare that they have no competing interests.

## References

[1] El-Abassi R, Singhal D, England JD (2014). Fabry’s disease. J Neuro Sci.

[2] Moon JC, Sachdev B, Elkington AG (2003). Gadolinium enhanced cardiovascular magnetic resonance in Anderson-Fabry disease. Eur Heart J.

[3] Moon JC, Sheppard M, Reed E (2006). The histological basis of late gadolinium enhancement cardiovascular magnetic resonance in a patient with Anderson-Fabry disease. J Cardiovasc Magn Reson.

[4] Gange CA, Link MS, Maron MS (2009). Utility of cardiovascular magnetic resonance in the diagnosis of Anderson-Fabry disease. Circulation.

[5] De Cobelli F, Esposito A, Belloni E (2009). Delayed-enhanced cardiac MRI for differentiation of Fabry’s disease from symmetric hypertrophic cardiomyopathy. AJR Am J Roentgenol.

[6] Sado DM, White SK, Piechnik SK (2013). Identification and assessment of Anderson-Fabry disease by cardiovascular magnetic resonance noncontrast myocardial T1 mapping. Circ Cardiovasc Imaging.

[7] Imbriaco M, Spinelli L, Cuocolo A (2007). MRI characterization of myocardial tissue in patients with Fabry’s disease. AJR.

